# Effects of perinatal exposure to daily maximum THI and THI fluctuations on serum total proteins and health of preweaned Holstein heifers raised in a dry climate

**DOI:** 10.1093/jas/skae218

**Published:** 2024-07-31

**Authors:** Diego Manriquez, Afrin Jannat, Ana Velásquez-Munoz, Pablo Pinedo

**Affiliations:** AgNext, Department of Animal Sciences, Colorado State University, Fort Collins, CO 80523, USA; AgNext, Department of Animal Sciences, Colorado State University, Fort Collins, CO 80523, USA; Departamento de Ciencias Veterinarias y Salud Pública, Universidad Católica de Temuco, Temuco 4780000, Chile; Department of Animal Sciences, Colorado State University, Fort Collins, CO 80523, USA

**Keywords:** climate, health, heifers, stress

## Abstract

The objective of this study was to assess the effects of the exposure to daily maximum and temperature-humidity index (**THI**) and to daily THI fluctuations (∆THI = maximum THI–minimum THI) at exposure periods comprising 2 d before birth to birth (−2 d), birth date (0 d), birth to 2 d of age (+2 d), and birth to 7 d of age (+7 d) on serum total proteins (**STP**), transfer of passive immunity (**TPI**), and the occurrence of scours and respiratory disease. A total of 841 Holstein heifer calves were retrospectively observed from −2 d until 65 d of age. Colostrum quality was assessed using a colostrometer to ensure a minimum globulin concentration of 52 mg/mL in the colostrum fed to the study calves. Two temperature and relative humidity sensors were installed at the calf yard. Maximum, minimum, and ∆THI values were obtained for each exposure period, and thermal exposure categories were defined as heat stress (**HS**: maximum THI > 70 units; non-HS: THI ≤ 70 units) and ∆THI (low < 20 units, medium ≥ 20 to ≤30 units, high > 30). The TPI was classified as poor (STP < 5.1 g/dL), fair (5.1 and 5.7 g/dL), good (>5.7 and 6.1 g/dL), and excellent (≥6.1 g/dL). Associations between the thermal exposure categories and the study outcomes were examined using ANOVA, logistic regression, and survival analyses. No differences in STP at −2 d were observed between HS and non-HS calves (6.83 ± 0.05 vs. 6.91 ± 0.05 g/dL), whereas HS-exposed calves at 0 d tended to have lower STP compared with non-HS calves (6.82 ± 0.05 vs. 6.92 ± 0.05 g/dL). Calves exposed to small ∆THI at 0 d had greater STP compared with calves exposed to medium ∆THI (7.00 ± 0.06 vs. 6.75 ± 0.05 g/dL). No association was found between HS, and ∆THI categories and the TPI category. The odds of scours were about 2 times greater in HS calves compared with non-HS calves at all exposure periods. In addition, HS calves were affected by scours between 9 and 15 d earlier than non-HS calves. Furthermore, high ∆THI favored the development of respiratory problems compared with medium and low ∆THI. Assessment of extreme THI values and THI fluctuations provides a research opportunity for assessing thermal stress in dairy heifer calves raised in dry climate.

## Teaser

Assessing the magnitude of daily maximum THI provides an opportunity to assess the intensity of thermal stress exposure on preweaned Holstein heifers. This research depicts the effects of heat stress THI, determined by maximum daily THI values, and THI fluctuations on the health of Holstein heifers during the preweaned period.

## Introduction

Adaptation to historic climate patterns and climate change is critical for the sustainability of the dairy industry. In the US, dairy operations are settled in various climates (Rotz et al., 2020). In concrete, the US can be subdivided into moist, dry, and marine climate regions (Kim et al., 2018; National Weather Service, 2022). Therefore, as milk is produced in all 50 states (Economic Research Service, 2022), understanding farms’ microclimate and its effects on animal health and performance is essential for mitigating thermal stress in dairy cattle.

The impacts of climate on physiologic signs of stress and dampened productivity and fertility are commonly measured using the temperature-humidity index (**THI**; Collier et al., 2006; Polsky and von Keyserlingk, 2017; Manriquez et al., 2018; Pinto et al., 2020), which combines air temperature and relative humidity (**RH**) to estimate the extent of environmental stress in dairy cattle (Kovacs et al., 2018). Lactating dairy cows are especially susceptible to heat stress (**HS**) due to elevated metabolism caused by milk production (Polsky and von Keyserlingk, 2017). On the other hand, little information is available regarding thermoregulation mechanisms under hot or cold conditions in young cattle (Roland et al., 2016). Regarding thermal stress conditions, there is an active discussion to determine THI thresholds associated with HS in preweaned dairy calves, including THI values associated with the onset of HS signs and the intensity, duration, and frequency of THI critical values associated with impaired health and performance (Kovacs et al., 2020; Dado-Senn et al., 2023). Current research suggests that calf discomfort, determined by increased respiratory rate and shade-seeking behavior to alleviate HS, can be observed at THI ≥ 72 units (Kovacs et al., 2018; Manriquez et al., 2018; Lemal et al., 2023) and that temperature surpassing 25 °C along with RH above 50% (THI ~ 72 units) cause clinical signs of HS in preweaned Heifer calves (von Keyserlingk., et al., 2013; Wang et al., 2020). Regarding critical THI exposure, it has been suggested that upper THI values of 69 units trigger changes in respiratory rate and rectal temperature. Additionally, significant HS signs can be observed at THI above 88 units (Kovacs et al., 2020; Dado-Senn et al., 2023). Thus, THI thresholds beginning at 69 units seem to be adequate for determining environmental HS conditions in dairy calves, although farm microclimate should be considered.

Due to the lack of consensus on THI thresholds as indicators of HS in dairy heifer calves, determining thermal stress zones accounting for specific farm’s climate areas is needed (Bohmanova et al., 2007), especially in dry regions where the weather is characterized by large day to night fluctuations in temperature and humidity (Doesken et al., 2022) as this variation might cause transient exposure to extreme THI that could be neglected if daily THI averages are used for assessing thermal stress in calves. In this sense, we hypothesized that the daily maximum and minimum THI can be categorized for evaluating thermal stress responses in preweaned dairy heifers and that these categories will be associated with the calf’s health status during the preweaned period. In consequence, the objective of this study was to assess the effects of HS, maximum daily THI values, and daily THI fluctuations (∆THI: maximum THI–minimum THI) around birth on serum total proteins (**STP**), the status of the transfer of passive immunity (**TPI**), and health of preweaned Holstein heifers raised under dry climatic conditions.

## Materials and Methods

### Study animals, farm, and calf management

About 841 Holstein heifer calves were retrospectively monitored from 2 d before birth until 65 d of age. The analysis included calves born between June 15, 2017, and June 3, 2018, in an organic dairy farm located in the eastern plains of northern Colorado, U.S. Holstein heifers born during this period were eligible to participate in this observational study. In the study farm, close-up cows were housed in collective calving pens with free access to an outdoor patio. Calves were separated from their dams within 30 min after birth and moved to the maternity facility. During the first hour of life, the navel was dipped with 7% solution of Iodine and 2.8 L of pasteurized pooled colostrum was administered using feeding bottles. The study farm determined colostrum quality using a colostrometer/hydrometer (Fleenor and Stott, 1980). Only colostrum with a globulin concentration of 52 mg/mL was fed to the study calves. Colostrum feeding was repeated at 3 and 8 h of life in maternity. Twenty-four hours after birth, calves were transferred to the farm’s calf-rearing facility and housed individually in polyethylene hutches (Agri-Plastics, Stoney Creek, ON, Canada), provided with a front yard of 2.25 m^2^, enclosed by a galvanized welded wire fence, and sand bedding in summer and straw bedding in winter. The calf-rearing facility was 2.1 km apart from the maternity and had a total area of approximately 174,000 m^2^ exposed to direct sunlight. At the calf yard, calves received 3.8 L of colostrum in a 10-hour interval 4 times. At 4 d of age, calves received 2.5 L of pasteurized milk every 12 h until 14 d of age. Additionally, from this day on, calves had access to a small amount of calf starter feed (16% Organic Calf Starter, Feedex Companies, LLC. South Hutchinsin, KS), which increased according to intake up to 2.3 kg/head/d until weaning. From days 15 to 49, calves received 3 L of milk every 8 h. At 50 d of age, milk was fed only in the mornings, and at 65 d of life, calves were weaned. Water was provided from day 1 in a plastic bucket (8 L) and filled twice per day. By farm protocol, dehorning was performed before 30 d of age using electrical cauterization under local anesthesia with veterinary supervision. Vaccination scheme included intranasal Inforce-3 (IBR, PI3, BRSV; Zoetis, Florham Park, NJ) at 1 d of age, Ultrabac-8 (*Clostridium chauvoei, C. speticum, C. haemoylticum, C. novyi, C. sordelli*, and *C. perfringens* type B, C, and D; Zoetis) at 21 d of age, Spirovac L5 (*Leptospira canicola, L. grippotyphosa, L. hardjo, L. icterohaemorrhagiae, and L. pomona* bacterin; Zoetis), and a booster of Inforce-3 and Ultrabac-8 at 42 d of age.

### Study design

A retrospective observational study was carried out using information from environmental temperature (T, °C), RH (%), and farm records (PCDart, Raleigh, NC). Since this was an observational study, researchers did not perform any procedure on the study animals and no institutional animal care and use committee approval was required. The study period extended from June 2107 to June 2018 and farm personnel were blinded to the study objectives during the data collection periods.

Two T/RH sensors (HOBO Pro v2, Onset Computer Corporation, Bourne, MA) were installed at 2 m from the ground between the hutch lines in the calf-rearing facility. A sampling rate of one reading every 15 min was used during the entire study period. Readings from both sensors were averaged and used to calculate daily maximum, average, minimum THI, and ∆THI values. Environmental THI was calculated using the equation reported by the NRC (1971): THI = (1.8 × T + 32) − ((0.55 − 0.0055 × RH) × (1.8 × T − 26)).

### Outcome variables and case definition

This study comprised STP (g/dL), the status of TPI (originated from calf’s STP concentrations), and the occurrence of scours or respiratory disease as the outcome variables. The on-farm records software provided information on STP, blood sampling age (in days), scours and respiratory disease events, dams’ parity, and dystocia.

According to farm protocol, STP was determined from blood serum collected by jugular venipuncture between 3 and 7 d of age. Due to operational constraints, the farm performed blood sampling once a week, therefore, variability in the blood sampling age was expected. This situation was handled by including blood sampling age as controlling variable, in which calves were classified as early (1 to 3 d), middle (4 to 7 d), or late (8 to 10 d) sampling age. Samples from calves older than 11 d of age were not considered for analyses as suggested by Wilm et al (2018) and Lombard et al (2020). Blood was collected in sterile tubes without anticoagulant and was allowed to clot before centrifugation. An optical digital refractometer was used to determine STP concentrations (Palm Abbe, Solon, OH). A TPI status category was created using STP values as poor (STP < 5.1 g/dL), fair (5.1 and 5.7 g/dL), good (>5.7 and 6.1 g/dL), and excellent (≥6.1 g/dL) as suggested by Lombard et al (2020). Scours and respiratory disease events were diagnosed, treated, and recorded by farm personnel following farm protocols. A scours case was diagnosed in the event of loose and watery feces with or without systemic symptoms. On the other hand, respiratory disease was diagnosed in the presence of persistent coughing, nasal and eye discharge, and unilateral or bilateral dropped ears (McGuirk, 2008). We considered only the first case of each disease as disease outcome. Calf age (in days) at diagnosis was considered for survival analysis.

### Explanatory variables

The HS and ∆THI categories at the exposure periods covering 2 d before birth date (−2 d), birth date (0 d), birth date to 2 d of age (+2 d), and birth date to 7 d of age (+7 d) were the main explanatory variables considered in this study. The effects of these variables on the study outcomes were analyzed in models separated by exposure period, which corresponds to the time frame where exposure to external THI was measured (−2, 0, +2, and +7 d).

For −2, +2, and +7 d, daily maximum and minimum THI and THI fluctuations (∆THI = maximum–minimum THI) were averaged by the length of each exposure period, therefore, a single value was obtained. Consequently, for all exposure periods, HS exposure was categorized as HS (maximum THI > 70 units) and non-HS (maximum THI ≤ 70 units). The THI cutoff for HS stress was selected based on the suggested critical THI values associated with HS signs (68 to 72 units; De Rensis et al., 2015; Kovacs et al., 2020; Dado-Senn et al., 2023) and previous work on the study farm evidencing that changes in behavior began at 70 units (Manriquez et al., 2018). The ∆THI was classified according to its quartile distribution as small (<20 units), medium, (≥20 to ≤30 units), and large (>30 units). Other covariates of interest were dam’s parity (primiparous [**PP**], lactation *n* = 1; multiparous [**MP**], lactation *n* ≥ 2), dystocia, and blood sampling age for STP determination.

The study farm recorded calving ease on a 5-point scale: 1 = spontaneous or normal, 2 = slight problem or no major intervention, 3 = needed assistance, 4 = considerable effort, or 5 = extremely difficult. For analysis purposes, a binary classification of dystocia (calving ease ≥ 3) was created according to Mee (2008).

### Statistical analysis

Data analyses were carried out in SAS 9.4 (SAS Institute Inc., Cary, NC). Daily maximum, average, and minimum THI were selected and calculated using PROC SQL. These data were grouped by date and merged with calf birth date to assign individual thermal stress categories at each exposure period. Descriptive analysis was performed using PROC FREQ and PROC MEANS. The relationship between external temperature and THI was assessed using Pearson’s correlation coefficient (PROC CORR) and linear regression (PROC REG). To assess the effects of THI exposure on the study outcomes, separate models were built for each exposure period. PROC MIXED was used to assess the association between STP and thermal stress categories. In the STP initial model, the covariates included were dam’s parity, dystocia, and blood sampling age. In addition, the interaction between thermal exposure categories and blood sampling age was evaluated. The TPI status category was analyzed as ordinal variable using proportional odds models (PROC LOGISTIC). Thus, the odds of having poor or fair vs. good or excellent TPI were estimated to perform multiple comparisons between the thermal stress categories. The covariates included in the TPI initial model were dam’s parity, dystocia, and STP sampling age category. PROC LOGISTIC was used to assess the association between scours and respiratory disease with the thermal stress categories. Covariates included dam’s parity, dystocia, TPI, and blood sampling age categories. In addition, logistic regression models were used to assess the association between the health outcomes and daily ∆THI as continuous predictor. Disease frequency was compared between the calvings occurred between the hot (Spring [March 1st to May 31st] and Summer [June 1st to August 31st]) and the cold (Fall [September 1st to November 30th] and Winter [December 1st to February 28th]) seasons using the Chi-square test. Time-to-event analyses (PROC LIFETEST) were performed to compare the diagnosis ages of scours and respiratory disease among the thermal stress categories. Survival curves and 95% Hall–Wellner confidence bands were generated for each strata of thermal stress categories. The Wilcoxon test determined significant differences in the survival functions between strata by exposure period. Statistical significance was determined at *P*-values < 0.05. Additionally, covariates with *P*-values ≤ 0.1 were kept in the models for confounder controlling.

## Results

### Descriptive statistics

We included 841 preweaned Holstein heifer calves in the analyses. A total of 196 (23.4%) and 642 (76.6%) calves were born to PP and MP cows, respectively. The seasonal distribution of calvings was Spring 11.9% (*n* = 100), Summer 41.5% (*n* = 348), Fall 28.2% (*n* = 236), and Winter 18.4% (*n* = 154). Dystocia was observed in 69 (3.5%) calvings.

**Figure 1.**
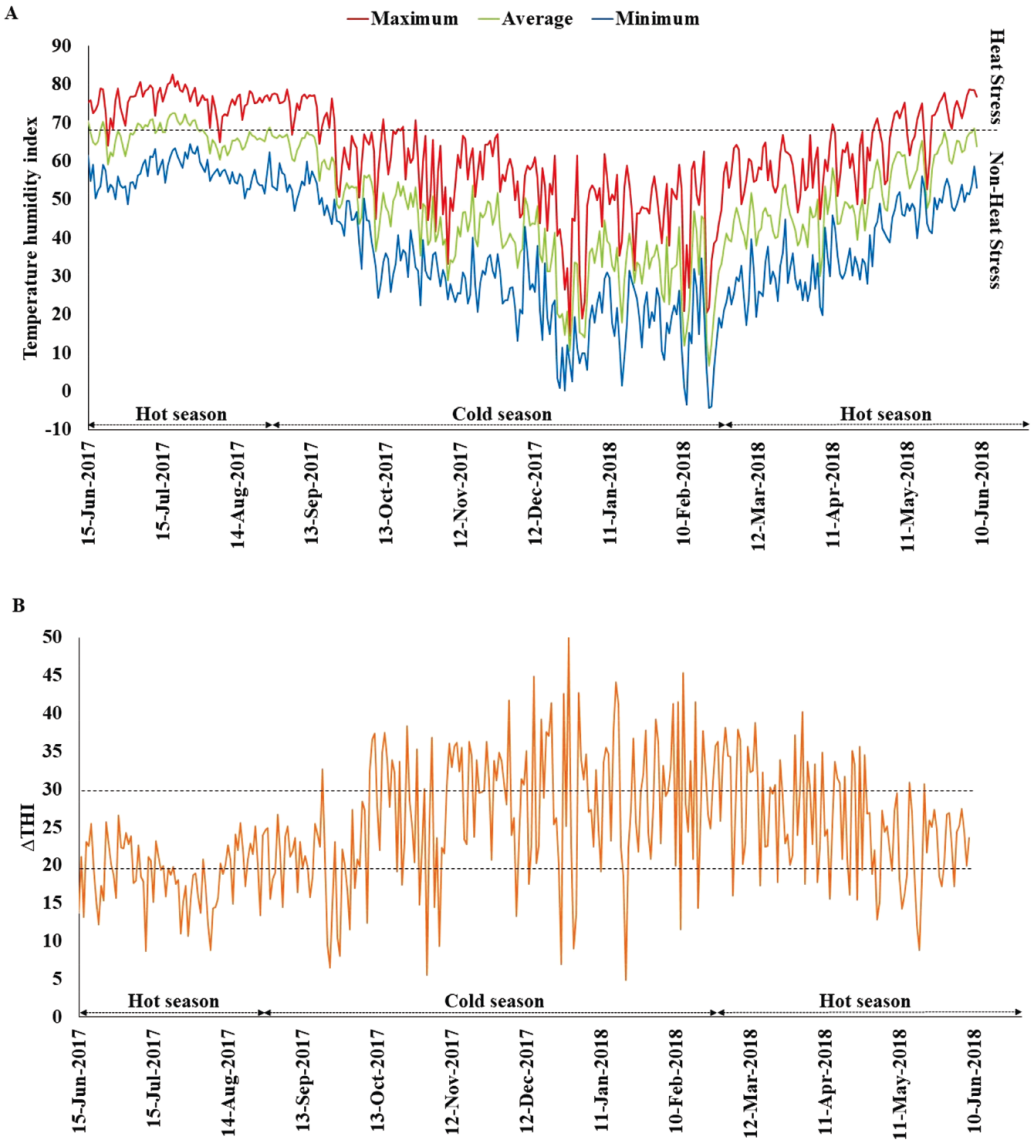
Daily minimum, average, and maximum THI (A) and daily THI fluctuation (∆THI, B) were recorded in the study farm during the study period. Thermal stress categories were defined as HS (THI > 70), non-HS (THI ≤ 70). Daily ∆THI was defined as low (< 20), medium (≥ 20 to ≤ 30), high (> 30), and hot season included Spring (March 1st to May 31st) and Summer (June 1st to August 31st) whereas the cold season included Fall (September 1st to November 30th) and Winter (December 1st to February 28th).

Figure 1A illustrates the daily maximum, average, and minimum THI during the study period. Additionally, in Figure 1A, HS was delimited following the thermal stress categorization. During the study period, temperature and THI were highly correlated (*r* = 0.99) and showed a positive linear association (*R*^2^ = 0.98; *P* < 0.001), therefore, THI was used as the main explanatory variable. Figure 1B shows the dynamics of ∆THI during the study periods throughout the hot and cold seasons. Figure 1A and B indicates that the study farm THI had great variation between maximum and minimum values within the same day. The first part of the hot season (Mid-March to Mid-June) was characterized by increasing maximum THI values and minimum values that fell below 50 THI units (average temperature 2.2 °C, Table 1). Conversely, the second half of the hot season was characterized by maximum THI values that mostly remained in the HS category with a few days below the non-HS threshold. When using maximum THI to determine HS exposure, the hot season had 100 (56.2%) and 78 (43.5%) d classified as HS or non-HS, respectively. On the other hand, we observed that the cold season had most of the maximum THI values under the non-HS category. During the cold season, when using maximum THI, a total of 20 (11.2%) and 161 (88.8%) d were classified in the HS and non-HS categories, respectively.

**Table 1.** Equivalence of daily maximum, average, and minimum THI exposure categories in terms of temperature and RH during the study period

	Temperature (°C)			Relative humidity (%)		
	Average (SD)	Minimum	Maximum	Average (SD)	Minimum	Maximum
Maximum THI						
HS (>70 units)	31.1 (3.4)	22.9	40	84.9 (9.1)	55.7	99.4
Non-HS (≤70 units)	12.8 (7.8)	−12.2	26.8	86.0 (10.4)	43.3	100
Average THI						
HS (>70 units)	8.95 (9.7)	−17.4	25.3	61.1	14.9	94
Non-HS (≤70 units)	17.9 (4.5)	7.4	25.3	56.66 (13.0)	23.9	90.3

THI: Temperature Humidity Index. HS THI level was not recorded when using minimum daily THI.

Table 1 shows the equivalence of the thermal stress levels in terms of temperature and RH during days classified as HS and non-HS using daily maximum and daily average THI values. Thus, Table 1 indicates that the average temperature of days classified as HS using maximum THI values was 31.1 °C (SD: 3.4 °C) and had a remarkably high average RH of 84.9% (9.1%). Table 2 shows the distribution of calves exposed to HS and ∆THI categories during the exposure periods considered in this study, which indicated a balanced distribution between the calves exposed to thermal stress and non-stress conditions. We observed a lower frequency of calves exposed to high ∆THI (range 13.4% to 18.7%) compared with medium and low ∆THI, which ranged between 32.5% and 54.1%. The mean (SD) of ΔTHI during the study periods was 24.8 (8.2) whereas the minimum, lower quartile, median, upper quartile, and maximum were 4.8, 19.2, 23.6, 30.9, and 50.1 ΔTHI units.

**Table 2.** Number (%) of calves classified into the HS and the daily THI change (∆THI) thermal exposure categories during the perinatal exposure periods considered in this study

Thermal zones	2 d before birth	Birth date	2 d after birth	7 d after birth
Maximum THI				
HS (>70 units)	415 (49.4)	414 (49.2)	392 (46.6)	414 (49.2)
Non-HS (≤70 units)	426 (50.6)	427 (50.8)	449 (53.4)	427 (50.8)
ΔTHI				
High (>30 units)	122 (14.5)	157 (18.7)	127 (15.1)	113 (13.4)
Medium (20 to 30 units)	375 (44.6)	344 (40.9)	384 (45.7)	455 (54.1)
Low (<20 units)	344 (40.9)	340 (40.4)	330 (39.2)	273 (32.5)

Concerning the study outcomes, the overall STP (SD) was 7.07 (0.89) g/dL and the overall distribution of the TPI categories was poor 0.5% (*n* = 4), fair 5.0% (*n* = 42), good 8.7% (*n* = 42), and excellent 85.8% (*n* = 719). The distribution of the blood sampling age category was early 40.9% (*n* = 330), middle 50.4% (*n* = 407), and late 8.8% (*n* = 71). Twenty-nine calves had blood samples collected at ages older than 11 d and 5 calves that did not have sampling age records were excluded from the analyses. During the observation period, a total of 507 (60.3%) calves were diagnosed with scours, whereas 567 (67.4%) were affected by respiratory disease. The median diagnosis age for scours was 15 d (95% CI: 14 to 17), whereas the median diagnosis age for respiratory disorders was 36 d (34 to 39).

### STPs and status of TPI

We evaluated the effects of HS and ∆THI on STP and TPI category during the exposure periods −2 and 0 d. We did not consider +2 and +7 d as blood sampling might have occurred before the completion of these exposure periods. The STP and TPI models included dam’s parity, blood sampling age category, and interaction terms between sampling age and the thermal stress categories. We did not observe differences between HS and non-HS calves in the exposure period −2 d on STP (6.83 ± 0.05 vs. 6.91 ± 0.05 g/dL; *P* = 0.14). On the other hand, HS calves at 0 d tended to have lower STP compared with non-HS calves (6.82 ± 0.05 vs. 6.92 ± 0.05 g/dL; *P* = 0.1).

Exposure to ∆THI category at −2 d did not impact STP (*P* = 0.35) as calves exposed to small (6.92 ± 0.06 g/dL), medium (6.83 ± 0.0 g/dL), and large (6.87 ± 0.08 g/dL) ∆THI levels had similar STP concentrations. Remarkably, the exposure to ∆THI during the day of birth had a significant effect on STP, in which calves exposed to low ∆THI had greater STP compared with calves exposed to medium ∆THI (7.00 ± 0.06 vs. 6.75 ± 0.05 g/dL; *P* = 0.0003), whereas high ∆THI-exposed calves tended to have greater STP compared with medium ∆THI-exposed calves (6.91 ± 0.08 g/dL vs. 6.75 ± 0.05 g/dL; *P *= 0.1). We did not observe STP differences between low and high ∆THI-exposed calves at 0 d (*P *= 0.58). Dam’s parity and blood sample age categories were retained as controlling variables in all STP models. Overall, calves born to MP cows tended to have greater STP compared with calves born to PP cows (6.94 ± 0.04 vs. 6.81 ± 0.07 g/dL; *P* = 0.06). We detected a significant overall main effect of blood sampling age category on STP, in which samples collected at early age resulted in greater STP concentrations compared with middle (7.36 ± 0.05 g/dL vs. 6.94 ± 0.05 g/dL; *P* < 0.0001) and late-age samples (6.32 ± 0.1 g/dL; *P* < 0.0001). In addition, middle-age samples had greater STP concentrations compared with late-age samples (*P* < 0.0001).

We considered the TPI category as an ordinal response, as greater TPI levels denoted increments of acquired passive immunity from colostrum consumption (Lombard et al., 2020). Therefore, we performed multiple proportional odds models (POM) to investigate the effect of HS and ∆THI exposure at −2 and 0 d. At these periods, we did not find significant differences on the odds of having poor or fair TPI between HS and non-HS calves. Similarly, we did not observe differences in the odds of TPI levels between the ∆THI exposure levels. The POM models had calf blood sampling age category as a significant covariate (*P < *0.0001), which indicated that calves sampled at early age had 7.00 (95% CI: 3.77 to 12.95; *P < *0.0001) times greater odds of having good or excellent TPI status compared with samples collected at late age and 1.86 (95% CI: 1.14 to 3.0.; *P < *0.0001) times greater odds of being in the good or excellent TPI status compared with middle sampling age. Finally, middle-age sampling had 3.75 (95% CI: 2.17 to 6.5; *P < *0.0001) times greater odds of having good or excellent TPI compared with late sampling.

### Health performance

We studied the effects of HS and ∆THI exposure during the exposure periods −2, 0, +2, and +7 d on the occurrence of scours and respiratory disease during the preweaning period. The TPI-adjusted odds ratios for scours and respiratory disorders are presented in Tables 3 and 4, respectively.

**Table 3.** Adjusted odds ratios (OR) for the occurrence of scours during the preweaned period of Holstein heifers exposed to HS or different levels of THI fluctuations during 2 d before birth to birth (−2 d), birth date (0 d), birth to 2 d of age (**+**2 d), or birth to 7 d of age (**+**7 d)

Thermal zones	−2 d		0 d		+2 d		+7 d	
	OR	*P* value	OR	*P* value	OR	*P* value	OR	*P* value
Maximum THI^1^								
HS vs. non-HS	2.14 (1.61 to 2.84)	<0.0001	2.23 (1.68 to 2.97)	<0.0001	1.97 (1.48 to 2.61)	<0.0001	1.96 (1.48 to 2.60)	<0.0001
ΔTHI^1^^,^^2^^,^^3^		0.006		0.006^3^		0.0003^3^		<0.0001
High vs. medium	0.88 (0.58 to 1.32)	0.53	0.58 (0.39 to 0.84)	0.005	0.56 (0.37 to 0.84)	0.005	0.71 (0.46 to 1.07)	0.1
High vs. low	0.52 (0.34 to 0.79)	0.002	0.56 (0.38 to 0.82)	0.003	0.43 (0.28 to 0.65)	<0.0001	0.34 (0.21 to 0.55)	<0.0001
Medium vs. low	0.59 (0.45 to 0.80)	0.0007	0.97 (0.71 to 1.32)	0.84	0.76 (0.56 to 1.03)	0.07	0.48 (0.35 to 0.67)	<0.0001

^1^Daily maximum and minimum temperature and humidity index (THI) and daily THI range (ΔTHI) were averaged for the exposure periods between 2 d before birth and birth, birth to 2 d of age, and birth to 7 d of age. The THI exposure was classified as HS (THI > 70 units) or non-HS (THI ≤ 70 units).

^2^ΔTHI was classified as small (<20 units), medium (≥20 to ≤30 units), or large (>30 units).

^3^TPI category removed from the model due to *P* value > 0.1.

Logistic regression models were adjusted by the status of TPI category.

**Table 4.** Adjusted odds ratios (OR) for the occurrence of respiratory disease during the preweaned of Holstein heifers exposed to HS or THI fluctuations during 2 d before birth to birth (−2 d), birth date (0 d), birth to 2 d of age (**+**2 d), or birth to 7 d of age (**+**7 d).

Thermal zones	−2 d		0 d		+2 d		+7 d	
	OR	*P* value	OR	*P* value	OR	*P* value	OR	*P* value
Maximum THI^1^								
HS vs. non-HS	0.72 (0.54 to 0.97)	0.03^2^	0.77 (0.58 to 1.03)	0.07^2^	0.70 (0.52 to 0.94)	0.02^2^	0.75 (0.56 to 1.00)	0.05^2^
ΔTHI^1^^,^^2^^,^^3^		0.02		0.004		0.05		0.003
High vs. medium	1.35 (0.84 to 2.15)	0.21	1.2 (0.78 to 1.84)	0.4	1.36 (0.87 to 2.15)	0.18	1.47 (0.91 to 2.38)	0.12
High vs. low	1.85 (1.15 to 2.95)	0.01	1.83 (1.20 to 2.79)	0.003	1.72 (1.10 to 2.72)	0.03	2.21 (1.34 to 3.68)	0.002
Medium vs. low	1.37 (1.00 to 1.88)	0.05	1.53 (1.11 to 2.10)	0.001	1.26 (0.92 to 1.72)	0.15	1.51 (1.10 to 2.07)	0.01

^1^Daily maximum and minimum temperature and humidity index (THI) and daily THI range (ΔTHI) were averaged for the exposure periods between 2 d before birth and birth, birth to 2 d of age, and birth to 7 d of age. The THI exposure was classified as HS (THI > 70 units) or non-HS (THI ≤ 70 units).

^2^ΔTHI was classified as small (<20 units), medium (≥20 to ≤30 units), or large (>30 units).

^3^TPI category removed from the model due to *P* value > 0.1.

Logistic regression models were adjusted by the status of TPI category.

Exposure to HS had a significant effect on the odds of scours during the preweaned period (Table 3). The odds of scours were around 2 times greater in calves exposed to HS compared with non-HS calves at all exposure periods considered in this study. In addition, we observed that calves exposed to low ∆THI had greater odds of scours compared with calves exposed to high and medium ∆THI (Table 3), which might also be related to the increased scour frequency as the hot season was characterized by lower daily ∆THI (Figure 1B) compared with the cold season (53.4% vs. 25.4%; *P* < 0.0001). In the HS models for scours, TPI was maintained as a controlling covariate (*P* values ranged between 0.06 and 0.1). On the contrary, the ∆THI models did not retain TPI as a controlling covariate, except for the 7 d exposure period (Table 3).

We observed a seasonal effect on the diagnosis of respiratory problems, which resulted in lower odds of respiratory disease when calves were exposed to HS at the exposure periods −2 and 2 d (Table 4). Moreover, we observed that ∆THI was a relevant risk factor for respiratory disease, in which calves exposed to high and medium ∆THI had significantly greater odds of developing respiratory problems compared with exposure to low ∆THI days during the perinatal period. This finding is relevant as days with high and medium ∆THI are observed across all seasons in the study farm (Figure 1B). The TPI status category was not retained as a controlling covariate in the HS models for respiratory disease. On the other hand, the odds for the respiratory diseases were TPI adjusted in the ∆THI models. When we studied the main effect of TPI on the odds of scours or respiratory diseases, we did not find a significant association between the levels of TPI and respiratory illness (*P *= 0.15).

As ∆THI was categorized using empirical data from the study farm climate, we analyzed the association between scours and respiratory disease and ∆THI as a continuous predictor to confirm the presence of the observed association and the directionally of it. For scours, we determined that one unit increase of ∆THI at −2 d was associated (*P* = 0.0001) with a 4% (95% CI: 3% to 7%) decrease on the odds of scours. On the other hand, for 0, +2, and +7 d, we observed a 3% (1% to 4%; *P* = 0.002), 4% (2% to 7%; *P* = 0.0001), and 7% (4% to 9%; *P* < 0.0001) decrease on the odds of being diagnosed with scours, respectively. Thus, we confirmed that a small ∆THI exposure favored the occurrence of scours in the study group. Regarding respiratory disease, one unit increase of ∆THI at −2d increased the odds of respiratory disease by 3% (1% to 6%; *P* = 0.008). At 0 d, one unit ∆THI increase was associated with 3% (1% to 5%; *P* = 0.004) increase on the odds of respiratory sickness, whereas we observed that the odds of respiratory increased by 4% (1% to 7%; *P* = 0.002) at +2 d and by 6% (3% to 9%; *P* = 0.0001) at +7 d when ∆THI increases by one unit.

**Figure 2.**
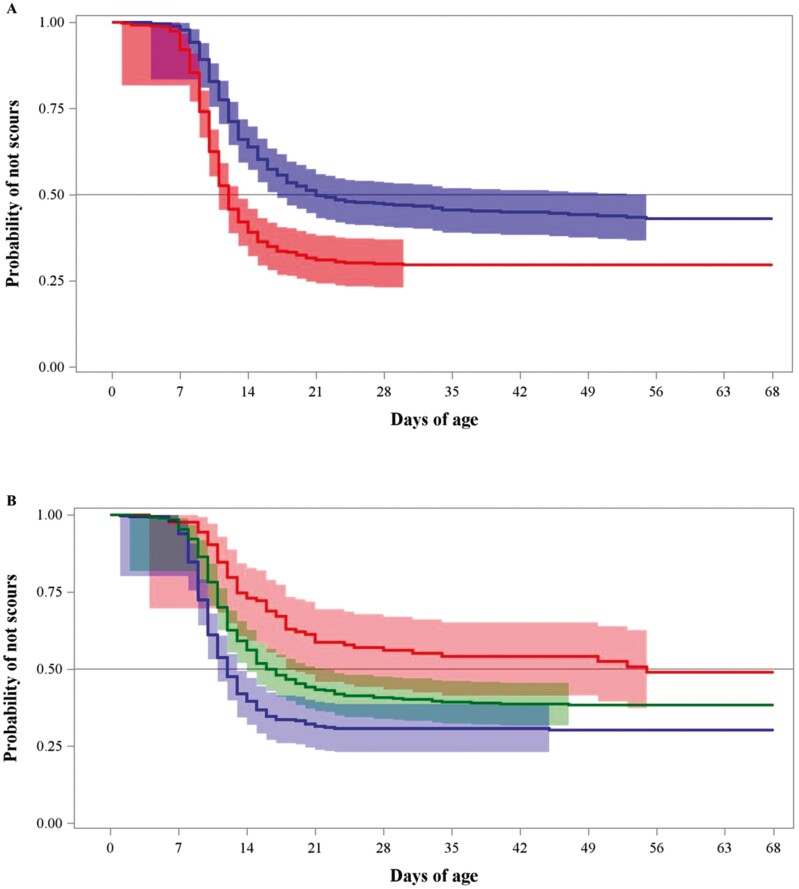
Kaplan–Meier survival probability curves of not scours occurrence during the preweaned period in Holstein heifer calves exposed vs. not exposed to HS during the first 2 d of age (A) or exposed to small, medium, or large daily THI fluctuation (∆THI) during the first 2 d of age (B). THI: Temperature and Humidity Index. Figure A: Calves exposed to HS (red; maximum daily THI > 70), non-heat stressed calves (blue; maximum daily THI ≤ 70). Wilcoxon *P* values for the difference between strata < 0.0001). Figure B: Calves exposed to small (blue, < 20), medium (green, ≥20 to ≤30), or large (red, >30) ∆THI. The 95% Hall–Wellner bands are provided for all curves.

**Figure 3.**
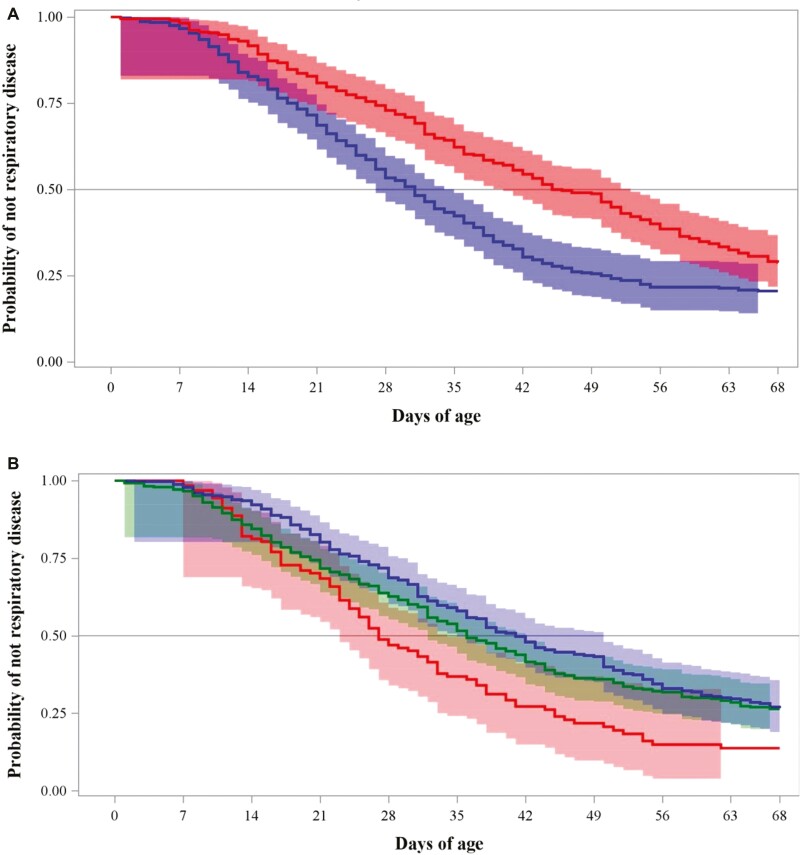
Kaplan–Meier survival probability curves of not respiratory disease occurrence during the preweaned period in Holstein heifer calves exposed vs. not exposed to HS during the first 2 d of age (A) or exposed to small, medium, or large daily THI fluctuation (∆THI) during the first 2 d of age (B). THI: Temperature and Humidity Index. Figure A: Calves exposed to HS (blue; maximum daily THI > 70), non-heat stressed calves (red; minimum daily THI ≤ 70). Wilcoxon P values for the difference between strata < 0.0001). Figure B: Calves exposed to small (blue, <20), medium (green, ≥20 to ≤30), and large (red, >30) ∆THI. The 95% Hall–Wellner bands are provided for all curves.

Regarding the time-to-diagnosis analyses, we observed significant differences in the survival functions of HS and ∆THI strata at all exposure periods. Median times of diagnosis of scours and respiratory diseases were lower in HS-exposed calves, respectively (Table 5). The median times for scours diagnosis in calves exposed to HS were very similar among the exposure periods, around 12 d of age. On the other hand, the median times of non-HS calves had scours diagnosis times ranging between 21 and 27 d of age, depending on the exposure period (Table 5). In addition, we observed differential patterns on the diagnosis ages for scours diagnosis determined by the levels of ∆THI exposure. Thus, calves exposed to small ∆THI were diagnosed with scours at earlier age (Table 5). In the case of the development of respiratory problems, we observed lower median diagnosis times for respiratory diseases in HS-exposed calves, 45 d of age (Table 5). In addition, we determined that calves exposed to high ∆THI were diagnosed with respiratory diseases at earlier ages compared with calves exposed to medium and high ∆THI (Table 5). All exposure periods resulted in relevant thermal stress assessment points and had similar survival curves. However, because of management and biological reasoning, we report the survival curves at +2 d as we considered this period critical for early life adaptation to climate conditions and for colostrum administration success. Figures 2 and 3 depict the survival curves for scours and respiratory disease diagnosis, respectively. The +2 d exposure period produced discriminable survival curves for the probability of scours and respiratory disease diagnosis, in which HS calves were affected by scours at earlier ages and non-HS calves were affected by respiratory disease at earlier ages. Moreover, THI fluctuations were a risk factor for early life disease development.

**Table 5.** Median time to diagnosis (95% CI) of scours and respiratory disease during the preweaned period of Holstein heifer calves exposed to daily maximum and THI fluctuation categories during 2 d before birth to birth (−2 d), birth date 0 d, birth to 2 d of age (+2 d), or birth to 7 d of age (+7 d)

Health outcome	Thermal zones	−2 d	0 d	+2 d	+7 d
Scours	Maximum THI				
	HS	12 (11 to 13)	12 (11 to 12)	12 (11 to 12)	12 (11 to 12)
	Non-HS	24 (19 to 27)	27 (19 to 35)	21 (18 to 37)	22 (18 to 47)
	ΔTHI^1^				
	High	25 (18 to 32)	34 (20 to 48)	55 (21 to 74)	50 (24 to 75)
	Medium	19 (15 to 24)	15 (13 to 17)	16 (15 to 20)	18 (15 to 21)
	Low	12 (11 to 13)	13 (12 to 14)	12 (11 to 13)	12 (11 to 12)
Respiratory disease	Maximum THI				
	HS	44 (38 to 50)	44 (39 to 50)	45 (42 to 52)	44 (41 to 50)
	Non-HS	32 (29 to 35)	31 (28 to 35)	31 (28 to 33)	31 (28 to 33)
	ΔTHI^1^				
	High	28 (24 to 32)	27 (23 to 32)	27 (24 to 33)	30 (24 to 33)
	Medium	35 (32 to 39)	36 (32 to 39)	36 (33 to 41)	36 (33 to 39)
	Low	42 (38 to 47)	42 (38 to 47)	41 (36 to 47)	43 (38 to 50)

^1^Daily THI fluctuation (ΔTHI: maximum–minimum THI) were averaged for the exposure periods between 2 d before birth, birth to 2 d of age, and birth to 7 d of age. The THI exposure was classified as HS (THI > 70 units) or non-HS (THI ≤ 70 units). ΔTHI was classified as small (<20 units), medium (≥20 to ≤30 units), or large (>30 units).

## Discussion

Thermal stress is an important factor determining growth, health, and productivity of domestic animals (Collier and Gebremedhin, 2015). In the context of climate change, utilizing on-farm climate measurements becomes critical to understand the effect of climate variation on animal health and performance and to identify critical levels of environmental thermal stress indicators, such as THI. The study farm climate is characterized by low RH, high altitude, abundant sunshine, infrequent rain and snow, and moderate to high wind movement. During the summer maximum temperatures usually reach between 35 and 38 °C, whereas the winter minimum temperatures commonly reach between −23 and −26 °C (Colorado Climate Center, 2003). Additionally, large daily and seasonal ranges in temperature are common, where temperature may fluctuate around 10 °C in a short time (Doesken et al., 2022). Therefore, we hypothesized that newborn calves are exposed to extreme and fluctuating THI conditions around birth and that there is enough variation in the THI exposure to evaluate the effects of thermal stress levels on the health of preweaned dairy heifers.

During the study period, high variation of temperature and RH levels were recorded, therefore, using THI values, which combine the effects of environmental temperature and air humidity (Kovacs et al., 2018), are relevant to determining the extent of thermal stress. Although it has been suggested that ambient air temperature (dry bulb temperature) is an optimal indicator of HS in continental climates (Dado-Senn et al., 2023), environmental data collected at the study farm data showed a great variability in RH, ranging from 8% to 100%, and a high linear association between external temperature and THI, therefore, we support the use of THI for assessing environmental thermal stress in the study farm climate. Thermal stress indicators for assessing negative impacts in calves and cows shall not be extrapolated to production systems without considering farm’s climate zone or the intensity of the thermal stress exposure (Dado-Senn et al., 2020b; Rotz et al., 2020). However, independently of local microclimates, there is universal concern about global surface temperatures that have already increased by 0.2 to 0.6°C since 2000 and are projected to increase by another 1.5 to 5.8 °C by the end of the century (IPCC, 2007). Additionally, this temperature increase is likely to influence regional patterns of precipitation (Sanderson et al., 2009). Therefore, strategies focused on specific climate zones will be crucial to mitigate thermal stress in dairy cattle with a vision of energetic and animal health sustainability.

Negative responses are exacerbated when animals are exposed to prolonged thermal stress, determined by THI levels associated with HS (Fu et al., 2022; Lemal et al., 2023).

Evidence suggests that THI values exceeding the range between 68 and 72 units are associated with the expression of physiological signs of HS and long-term negative effects on production, fertility, and immune response in dairy cows (Takahashi, 2012; Polsky and von Keyserlingk, 2017; Bagath et al., 2019; Pinto et al., 2020; Tao et al., 2020; Lemal et al., 2023). In response to this evidence, several heat abatement systems have been implemented for dairy cows, including shade systems, fans, sprinklers, and barn designs (Dado-Senn et al., 2020b; Davidson et al., 2021). Compared to mature dairy cattle, less information exists on tolerable thermal zones and thermal abatement systems for in-hutch Holstein heifer calves. Nonetheless, it has been suggested that dairy calves are more resistant to HS due to lower production of metabolic heat and increased heat dissipation efficiency (Dahl et al., 2019; Wang et al., 2020). Kovacs et al (2020) determined upper critical THI values associated with changes in rectal temperature, ear temperature, heart rate, and salivary cortisol starting at 78 units. In contrast, our study performed a retrospective evaluation in health outcomes that occurred after HS in specific exposure periods using a daily maximum THI of 70 units as a HS threshold. Using this approach, we observed that HS calves had greater odds of scours, supporting the use of a THI of 70 units as a critical value for HS in newborn calves raised in dry climate. Regarding THI thresholds for cold stress in calves, one study defined CS conditions for calves at 5.3 ± 1.1 °C by testing the effect of supplemental fat on calf performance (Litherland et al., 2014). However, no specifications of humidity or climate zones were provided. Other reports have established an average of 5 °C and 68% RH as cold conditions for calves (Roland et al., 2016), while others have induced transient CS at 10 °C in cooling chambers (Silva et al., 2021). Therefore, THI thresholds for determining environmental CS conditions are still matter of debate. Although THI is widely used for HS evaluation, it has been used for CS evaluation in dairy cattle (Mader et al., 2010; Fu et al., 2022). Nonetheless, accurate determination of environmental HS and CS conditions requires the development of more comprehensive indexes that incorporate other parameters such as wind speed and solar radiation (Mader et al., 2010), which may amplify warm and cold weather perceptions (Becker et al., 2021).

In our study, the exposure to HS during the perinatal period was associated with increased odds of scours but not with increased odds of respiratory disease. These results are opposed to the study performed by Louie et al (2018) that determined that maximum THI and the occurrence of BRD are positively associated. Conversely, we observed that the main factor associated with respiratory disease was high ∆THI, in which a large daily variation between maximum and minimum THI levels increased the odds of respiratory disease. On the other hand, we observed that HS and low ∆THI were associated with increased odds of scours. Thus, the combined effect of extreme temperature fluctuation can be incorporated as an assessment tool for the effect of environmental conditions on calf health. In our study, the ∆THI categorization was based on the empirical quartile distribution as, to the best of our knowledge, there are no recommended ∆THI thresholds for dairy calves raised in dry climate, therefore our ∆THI categorization should be further researched. Nonetheless, we concluded that ∆THI was a major factor in scours and respiratory disease diagnosis, in which small ∆THI favored the occurrence of scours whereas calves exposed to high ∆THI were more susceptible to respiratory disease. Thus, variation in climate conditions within the same day appears to be an important factor for the health of calves raised in outdoor hutches.

Calves born to cows exposed to HS during the dry period and fed with their dams’ colostrum still have compromised passive and cell-mediated immunity compared with calves born to cooled cows, although the singular effects of a heat-stressed dam and colostrum quality effect are not fully elucidated (Monteiro et al., 2016; Dado-Senn et al., 2020b). Nonetheless, we were limited to explore the effects of thermal stress on colostrum quality and the subsequent TPI as study farm used a qualitative method (colostrometer/hydrometer) to assess colostrum quality and pooled colostrum for feeding. In addition, only colostrum having at least 52 mg/mL of globulin was fed to the study calves. Considering this limitation, our study showed that HS-exposed calves tended to have lower STP, which agreed with the results presented by Monteiro et al. (2016), in which in-utero HS calves had lower serum concentration of IgG and consequently lower TPI compared with TN exposed calves. These results support the idea that in-utero HS reduces passive immune transfer regardless of colostrum source (Dahl et al., 2020). However, no data from the THI exposure during the dry period or calf’s serum IgG were available, which limits our conclusions about the effect of prenatal thermal stress and TPI. Further research using extreme THI daily values and ∆THI is needed to elucidate the effects of thermal stress intensity and THI fluctuations on successful TPI. Notably, we observed a large proportion of calves with excellent TPI (85.8%) compared with the excellent TPI proportion reported in the United States (35%; Lombard et al., 2020). Therefore, the differences found in STP between thermal stress categories should be used with caution as the occurrence of measurement error or hyperproteinemia cannot be tested in our dataset, which represents a study limitation. It has been suggested that hot conditions may cause calf dehydration, which might alter the concentration of STP. Dehydration causes an acute increase in albumin, which makes up to 62% of the serum proteins (Busher, 1990). Therefore, calves affected by health disorders, associated with dehydration, at the time of STP screening may show hyperproteinemia and reveal acceptable levels of STP. Nonetheless, we observed lower STP in HS calves, which suggests that thermal stress has a profound effect on calf’s ability to acquire TPI or on colostrum intake (Dado-Senn et al., 2020a).

## Conclusions

There was no significant association between HS exposure and STP and TPI, although HS calves tended to have lower STP. Exposure to small ∆THI (<20 units) at 0 d resulted in higher STP concentration compared with medium ∆THI (20 to 30). Calves exposed to HS (THI > 70 units) during the perinatal period had impaired health and were diagnosed with scours at an earlier age. Exposure to small ∆THI increased the odds of scours at earlier ages and large ∆THI increased the development of clinical symptoms of respiratory disease. Extreme daily THI values and THI fluctuations can be used to classify the level of thermal stress in newborn dairy calves raised in dry climate and evaluate subsequent effect on calf performance during the preweaned period.

## Abbreviations

ADG: average daily gain
HS: heat stress
LCT: low critical temperature
RH: relative humidity
STP: serum total protein
THI: temperature-humidity index
TPI: transfer of passive immunity
